# Evaluation of Ecotoxicity in Waste Leachate: A Current Status of Bioassay and Chemical Analysis

**DOI:** 10.3390/toxics13030198

**Published:** 2025-03-10

**Authors:** Lia Kim, Jin Il Kwak, Youn-Joo An

**Affiliations:** Department of Environmental Health Science, Konkuk University, 120 Neungdong-ro, Gwangjin-gu, Seoul 05029, Republic of Korea; kokoria12@konkuk.ac.kr (L.K.); kjigod@konkuk.ac.kr (J.I.K.)

**Keywords:** waste, leachate, ecotoxicity, bioassay

## Abstract

As global waste generation increases, waste toxicity has become a significant global issue. Among various hazardous properties, ecotoxicity refers to the risks that waste may pose to the environment. It is evaluated through aquatic bioassays to assess the effects of leaching contaminants, as well as through soil assessments where waste is buried. To clarify these issues, this study collected waste leaching methods from international organizations and various countries and analyzed case studies of bioassays for waste leachates. The criteria for determining the ecotoxicity of waste leachates were also reviewed, revealing inconsistencies in leaching methods across the European Union, the United States, Canada, and Asian countries. Additionally, various bioassays were applied to assess waste leachates, further contributing to inconsistencies. Given these variations, we recommend developing a unified leaching method, standardized bioassays, and consistent criteria for assessing the toxicity of waste leachates.

## 1. Introduction

The growing volume of waste generated globally poses significant environmental challenges, and its proper management is essential to mitigate adverse impacts on ecological systems [1,2]. Waste, defined as substances or objects that are discarded or intended to be discarded [3,4,5], can take many forms, including municipal, industrial, agricultural, and hazardous waste [3,4,5,6,7]. As waste production increases, the system for waste classification and management has become crucial to preventing harm to the environment [4].

With the goal of identifying materials that may pose risks to human health and ecosystems [3,4,5,6,7,8], waste classification is typically based on a combination of physical, chemical, and biological properties [3,4,8]. Among the various properties used to assess the potential harm of waste, ecotoxicity has become a key factor in identifying waste that poses risks to the environment [7,8].The ecotoxicity of waste refers to the immediate or delayed risks to ecosystems, as well as the potential hazards posed by waste buried in soil and toxicants leaching due to contact with intended or unintended moisture [4,9,10]. As such, identifying ecotoxic waste is an essential step in waste management and disposal.

In the European Union (EU), waste is classified through frameworks such as the European List of Waste [8], the Waste Framework Directive [3], and EU waste law [11]. These frameworks provide guidelines for classifying waste based on 15 hazardous properties (HP), including ecotoxicity (HP14). The regulatory framework recommends determining the ecotoxicity of waste using the following three categories: bioassays, chemical classification, and hazard index [3,4,8,11]. This framework guides member states in assessing whether waste is hazardous and requires the implementation of testing protocols to evaluate aquatic or terrestrial toxicity [4]. However, the methods and criteria for testing ecotoxicity in waste leachates differ across countries. For instance, Germany, Italy, and France have their own protocols for assessing the ecotoxicity of waste, including leachates, using aquatic organisms [12,13,14,15]. However, these approaches lack standardization, leading to inconsistencies in how waste is classified and managed. These variations pose a challenge to the harmonization of waste management both within Europe and globally.

The United States Environmental Protection Agency (USEPA) also has its own waste management program, the Resource Conservation and Recovery Act (RCRA), which outlines frameworks for managing both hazardous and non-hazardous solid waste [2]. Unlike the EU’s waste classification system, the USEPA defines four characteristics—ignitability, corrosivity, toxicity, and reactivity [16,17,18,19]—without a specific category for ecotoxicity. Based on the quantitative and qualitative analysis of chemicals and physicochemical properties [2,16,17,18,19], bioassays for waste leachates are not considered in the USEPA system.

Furthermore, other countries, including Canada, Japan, and Korea, do not incorporate ecotoxicity as a defined property for waste categorization [6,20,21]. In these regions, waste is primarily categorized based on physical and chemical properties, with little consideration of the indirect environmental toxicity of waste through moisture contact. These differences highlight the varying global approaches to waste classification and underscore the need for international standardization in ecotoxicity testing for waste leachates.

Given these disparities in determining the ecotoxicity of waste leachates, the need for a standardized protocol has become apparent. Thus, this paper reviews country-specific leaching methods and ecotoxicity criteria for waste, including those established by international organizations, as well as case studies on waste leachates that conducted bioassays. This research aims to provide recommendations for a harmonized leaching method and criteria for determining the ecotoxicity of waste leachates.

## 2. Standard Methods for Waste Leaching

The leaching of waste materials is a critical process for understanding their potential environmental impacts, particularly regarding the risk of contamination to soil and water. Several international standard methods have been developed to guide the testing of leachates from both soil and waste materials [22,23,24,25,26,27,28,29]. These standards differ in their methodologies and objectives, reflecting the unique characteristics of the materials being assessed and the ecotoxic concerns in different regions. The International Organisation for Standardisation (ISO) provides a set of guidelines for the leaching of soil and soil-like materials, aimed at ecological and chemical testing [22,23,24,25]. The European Committee for Standardisation (CEN) has developed more specific leaching methods focused on waste materials [26,27,28,29], considering factors such as sample size, weight, and solid/liquid ratios. The USEPA focuses on hazardous waste classification based on leachate testing, using the Toxicity Characteristic Leaching Procedure (TCLP) [16]. Meanwhile, other countries, such as Korea and Japan, have their own leaching methods [20,21], which are comparable in several respects but differ in detail, such as solvents or filtration procedures.

### 2.1. ISO Methods

The International Organization for Standardization (ISO) provides four methodologies for leaching soil or soil-containing materials [22,23,24,25]. These methods aim to formulate leachates for ecological and chemical testing of soil and similar materials. The ISO 21268-1 and ISO 21268-2 are batch tests, which use different liquid-to-solid ratios (2 L/kg and 10 L/kg, respectively) [22,23], while ISO 21268-3 is an up-flow percolation test designed to simulate the movement of water through soil materials and pores [24]. However, this study focused on waste, so ISO 21268-3 was not discussed in detail, as shown in Table 1. ISO 21268-4 considers the effect of pH on the leaching procedure for soil or soil materials through the addition of an acid or base [25]. As the ISO 21268 series is primarily concerned with soil leaching, a method for waste eluate preparation has not been established by ISO. Nevertheless, the previous studies have applied ISO 21268-2 to materials with similar properties to soil, such as char residues, plastic wastes, used tires, and pine forestry biomass [30,31,32].

### 2.2. EU CEN Methods

The CEN has developed specific leaching methodologies for waste, offering four different procedures [26,27,28,29]. Among them, CEN 12457-1, 12457-2, and 12457-4 are one-step batch tests that differ in sample size, weight, and solid-to-liquid ratios [26,27,29]. CEN 12457-3 comprises a two-stage batch test, where the first step uses a solid-to-liquid ratio of 1:2, and the second has a cumulative ratio of 1:10 [28]. Prior studies for waste ecotoxicity tests, fly ashes, photovoltaic panels, and co-combustions of meals have been conducted by using the CEN 12457-2 method [33,34,35,36,37,38,39,40,41,42,43,44]. This approach is mentioned in French waste hazard characterization guidelines [12,13]. The ISO and CEN leaching methods have a common point in that the liquid-to-solid ratios are 2 L/kg and 10 L/kg [22,23,26,27]. The leaching conditions following the methods suggested by CEN are presented in Table 2 [26,27,28,29].

### 2.3. US EPA Methods

The US EPA classifies hazardous waste based on four characteristics: ignitability, corrosivity, reactivity, and toxicity [16,17,18,19]. To evaluate whether the waste is hazardous, leachates from solid waste are tested using Method 1311, which outlines the Toxicity Characteristic Leaching Procedure (TCLP) [16]. This procedure requires samples with a sieving diameter of 9.5 mm and a weight of 100 g. Key differences with other standard methods include the use of acetic acid and sodium hydroxide as solvents and a solid-to-liquid ratio of 1:20. Additionally, the temperature and filtration methods differ. Due to the solvent composition, TCLP eluates have a lower pH compared with CEN eluates [29]. The Synthetic Precipitation Leaching Procedure (SPLP) is similar to the TCLP, but it uses a solvent based on sulfuric and nitric acid. In addition to the TCLP and SPLP methods, the Extraction Procedure (EP) and Multiple Extraction Procedure (MEP) are also available under the USEPA [18,19] (Table 3). Some states in Canada also use the TCLP method to derive leachate toxic chemicals from solid waste [45,46].

### 2.4. Korean and Japanese Methods

Korea’s Wastes Control Act categorizes waste into the following four types: household waste, industrial waste, designated waste, and medical waste [21]. Among these, designated waste consists of hazardous materials. Leachates are obtained by following the general test method defined in the Waste Official Test Standard. The prepared sample size ranges from 0.5 mm to 5 mm, and the leaching process is carried out by using a hydrochloric acid-based solvent for six hours. The ratio of solid to liquid was 1:10, and the leachate was filtrated by using a 10 µm pore size glass fiber filter. Japan also has its own standard leaching method, and it has similar leaching conditions to those of the Korean method, except for the solvent for heavy metals [20,47] (Table 4).

## 3. Characterization of Ecotoxicity on Waste Leachates

The ecotoxicity of waste leachates is assessed using bioassays, which provide direct evidence of potential risks to various aquatic organisms [7,8,12,13,14,15]. These bioassays play a crucial role in evaluating the ecological impacts of waste leachates, as they measure biological responses rather than relying solely on chemical toxicity. Various standardized bioassays are employed to evaluate the toxicity of waste leachates, using aquatic species such as *Aliivibrio fischeri*, *Brachionus calyciflorus*, *Ceriodaphnia dubia*, *Danio rerio*, *Daphnia magna*, *Lemna minor*, *Poecilia reticulata*, *Raphidocelis subcapitata*, *Sinapis alba*, and *Xenopus laevis* [48,49,50,51,52,53,54,55,56]. These organisms represent different trophic levels in aquatic ecosystems, providing comprehensive insights into the ecotoxicity of waste leachate. By reviewing the regulatory thresholds of various countries, mainly focused on European countries, a common threshold value can be deduced to classify ecotoxic waste leachates based on bioassay results. Additionally, the analysis of case studies that conducted bioassays to assess waste leachates using aquatic organisms can help identify the main bioassays currently in use.

### 3.1. Criteria for Determining the Ecotoxicity in Waste Leachates

The ecotoxicity of waste characterization, defined as HP14, is categorized as the following three risk-progressive groups: acute hazard, chronic aquatic hazard, and hazard to ozone layer [3,4,7,8]. The chronic aquatic hazard category is further divided into four subcategories based on the potential for long-term adverse effects on aquatic life [7]. To assess these hazards, different assessment methods are used in each country. Especially for European countries [12,13,14,15], waste leachate is used for HP14 characterization, which indicates ecotoxicity. Leachates formulated based on the standard leaching methods from ISO or CEN [21,22,23,24,25,26,27,28,29] are subjected to both chemical analysis and ecotoxicity tests. However, Austria and Belgium rely only on chemical analysis of waste leachates, whereas the Czech Republic and Spain include ecotoxicity tests using aquatic organisms [8,13].

In France and Germany [12,13,14,57], the evaluation protocol for the waste ecotoxicity property “HP 14” is being progressively implemented using both aquatic and terrestrial bioassays [12,14,57]. In addition, bioassays using different test species directly derived from solid waste are being performed. This protocol with reference values has been suggested by France [12,57]. In this procedure, waste leachates are formulated based on the guideline of the CEN 12457-2 method [27]. Subsequently, ecotoxicity tests are performed if the chemical characterization of waste leachates does not exceed the concentration limits. The results of bioassays for waste leachates can be the next hurdle in the final determination of ecotoxicity, as they are compared with the proposed threshold values [12,13,14,15,57].

With regard to the initial development of the ecotoxicity characterization methods, some studies carried out multi-species ecotoxicity tests with various types of wastes [12,33,58]. Leachate bioassays were conducted in acute and chronic tests with common aquatic species such as *Vibrio fischeri*, *Brahoionus calyciflorus*, *Pseuokirchneriella subcapitata*, and *Daphnia manga*. In ecotoxicity characterizations, pH of the waste leachate is regarded as one of the important properties of waste; further, the CEN 14735 guidelines indicate that the pH adjustment is only acceptable when the pH exceeds the value necessitated for the survival of the test species [7,14,59]. The toxicity values are calculated as 50% or 20% of the effective concentration (EC_50_ and EC_20_), and the lethal concentration is regarded as 50% (LC_50_); subsequently, these values are compared to the suggested reference values to characterize the ecotoxicity. Some studies have calculated toxic units (TUs) and performed characterization on the basis of the previous methods that have been performed [13,60,61,62,63].

To standardize the characterization methods, European countries have made significant efforts to designate guidelines for defining the standard test methods and concentration limits for certain test species. Moser and Römbke et al. [63] comprehensively evaluated the procedures for waste leachate toxicity tests; their interpretations of the test results mentioned that the batteries of aquatic tests should be used to characterize waste leachates, followed by examining the correlation of the dose–response data. If the toxicity values are under the limit values, bioassays with terrestrial species should be applied; this has been permitted in France, Germany, and Italy in the past. Waste can be characterized as “not ecotoxic” in the case that both values of the aquatic and terrestrial toxicities are under the concentration limit. The Italian waste ecotoxicity test method guideline followed the protocol and threshold standards of France [12,13,57]. Ecotoxicity tests were carried out on waste leachates to classify the HP 14 characteristics with regard to different test species by using the ISO test guidelines. Tammaro et al. [36] applied Italian waste standard toxicity values for assessing 38 photovoltaic panels, and it was observed that 25 of the thin films among these panels showed non-ecotoxicity. This application of ecotoxicity assessment of wastes has been recommended in Italy but has not been currently applied for waste characterization [13].

Germany suggested the use of waste ecotoxicity assessment methods [14] that were carried out for aquatic and terrestrial tests to perform ecotoxicity analysis by using ISO standard methods [48,50,51]; in these methods, the test species should comprise three trophic levels, namely, producers, consumers, and decomposers. Results of the tests conducted by Pandard and Römbke [64] are being evaluated with the proposed concentrations. These standard values suggested by Pandard and Römbke [64] have been applied in recent studies [65,66,67]. Furthermore, ISO/DS 17616 selected biological tests for ecotoxicological assessment in contaminated soils or soil materials using *Aliivibrio fischeri*, *Raphidocelis subcapitata*, *Daphnia magna*, *Lemna minor*, *Ceriodaphnia dubia*, and *Brachionus calyciflorus* [48,49,50,51,53,55,68]. The suggested values for the lowest ineffective dilution (LID) and significant biological effect criteria [27,69] can be applied as reference thresholds, following a similar protocol for determining ecotoxicity using aquatic organisms, although it is confined to the determination of soil contamination [68].

All the details about waste leachate bioassays in European countries are presented in Table 5. Finland, Italy, and the United Kingdom currently do not apply the ecotoxicity tests on waste leachates due to the absence of the discouragement provided by the threshold values despite the existence of recommendations of conducting bioassays. There are various kinds of toxicity values, test species, and units that cannot undergo waste classification due to the existing differences. These problems need to be addressed by standardizing the classification threshold values with the same units and test species, regardless of the location of the bioassays being conducted [13].

### 3.2. Case Studies of Bioassay for Waste Leachates

We further reviewed the case studies of aquatic bioassays that have been conducted to determine the ecotoxicity of waste by generating the waste leachates mentioned in Section 2 [30,31,32,33,34,35,36,37,38,39,40,41,42,43,44,59,66,67,70,71,72,73,74]. Table 6 presents a detailed review of bioassays conducted to assess the ecotoxicity of waste leachates, highlighting the solid waste types, leaching methods, and test species used for bioassays. First of all, the waste types covered a wide range of industrial, municipal, and emerging wastes, reflecting the growing complexity of waste management challenges. In addition to the traditional waste types, such as coal fly ash and slags from iron or steel production [30,31,32,33,34,59], new waste categories, including photovoltaic panels, fire retardant coating systems, and cigarette butts, show the increasing need for ecotoxicity assessment of technologically advanced materials or wastes today [32,36,66,72].

As the leaching method for bioassays, EN 12457-2 was chosen as the primary leaching method [33,34,35,36,37,38,39,40,41,42,43,44], making it suitable for determining waste ecotoxicity. Other leaching procedures, such as TCLP, ISO/TS 21268-2, and CEN/TS 16637-2, are also used depending on the specific characteristics of the wastes and environmental exposure scenarios [30,31,32,34,70]. One important point is that the pH of waste leachates is a critical factor affecting ecotoxicity, and the effects of pH must be considered when performing bioassays.

Diverse bioassays have been conducted to evaluate the ecotoxicity of waste leachates, providing insights into the impacts at different ecological levels, from producers to consumers [48,49,50,51,52,53,54,55,56]. The predominant use of *Aliivibrio fischeri*, *Daphnia magna*, and *Raphidocelis subcapitata* indicates luminescence inhibition, immobilization of invertebrates, and algal growth inhibition as key tests for ecotoxicity assessments [48,50,51]. By using *Ceriodaphnia dubia*, embryos of *Danio rerio*, and *Lemna minor*, the ecotoxicity of waste leachates can cover a wide range of potential risks to ecological systems, including the effects on higher trophic level organisms [55,72]. Additionally, the variation in the selection of test species reflects the differences in national or international regulatory guidelines, as some countries prioritize certain species based on environmental relevance and legal requirements [12,13,14,49,53,55].

The variation in test methods between case studies and protocols suggested by each country highlights the importance of harmonizing bioassays and regulatory thresholds for determining the ecotoxicity of waste leachates to ensure consistent and reliable waste classification across regions. By integrating standardized bioassays into regulatory frameworks, authorities in each country and organization can improve the identification of hazardous wastes and effectively minimize the environmental risks induced by waste contamination.

### 3.3. Characterization of Waste Leachates by Chemical Analysis

In cases where bioassay-based criteria have not been established to determine the ecotoxicity of waste leachates, as is the case with the US EPA, Canada, Japan, and Korea, the toxicity of waste leachate is determined based on the concentration of contaminants (Table 7) [5,6,16,20,21,45,46,47]. However, the chemical concentration-based determination of toxicity is not an alternative method for characterizing the ‘ecotoxicity’ of waste leachates.

The US EPA uses the TCLP method for assessing leaching toxicity, with the waste classified as hazardous if the contaminant level exceeds the regulatory limits [16]. Canada partially adopts the US EPA method (TCLP), including the bioassay-based toxicity assessment, such as LC_50_ values for rainbow trout [45,46]; this indicates that bioassays provide an additional layer of environmental protection beyond the threshold of chemical concentrations [45,46].

The Ministry of Environment (MOE) of Korea follows a similar approach to the US EPA, with the Enforcement Rule of the Waste Control Act specifying hazardous substances and their concentration limits [21]. Moreover, the MOE of Japan emphasizes the prevention of groundwater contamination from effluent-derived contaminants for municipal solid waste and industrial waste [20]. However, Japan does not officially integrate bioassays into the category of ‘ecotoxicity’ [20,47]. These countries lack a formalized framework for assessing the ecotoxicity of wastes through biological testing, highlighting the differences in regulatory strategies among them.

## 4. Discussion

The determination protocol of ecotoxicity on waste leachates varies across the countries and organizations, primarily depending on whether chemical analysis and bioassays are performed. In European countries, bioassays are actively used to directly evaluate the environmental impacts of waste leachates [12,13,14,15], whereas countries such as the United States, Canada, Japan, and Korea rely on the threshold concentrations of contaminants for determining the toxicity [16,21,45,47]. These differences stem from distinct environmental policy and regulatory frameworks, highlighting the lack of harmonized processes and criteria for ecotoxicity decisions in waste leachates.

Chemical analysis provides clear and quantitative criteria for waste toxicity [16,21,45,47], but this approach would have potential risks that cannot reach the interactive and cumulative effects of waste contaminants in real environmental conditions. In contrast, the bioassay directly measures the impacts of waste leachate using aquatic organisms, offering a more comprehensive approach. Despite their advantages, the bioassay for waste leachate also has challenges, such as variability in leaching methods, test species selection, and different criteria by country, which can lead to inconsistent classification even in the same waste types. Consequently, the absence of a globally harmonized framework for waste leachate ecotoxicity needs to be addressed.

Thus, we suggest that the international standardization effects should be promoted to harmonize the waste ecotoxicity assessment. Organizations such as ISO, CEN, and US EPA can collaborate to establish a unified leaching method for wastes, as well as the selection of bioassays. By setting the common criteria for determining the ecotoxicity of waste leachates, the countries and organizations can maintain regulatory consistency, allowing flexibility for specific national regulations.

The use of bioassays can be expanded and refined; we can enhance the reliability of waste leachates in the aspect of ecotoxicity via further standardization and use of multi-species bioassays, including both aquatic and terrestrial ecological receptors. The development of globally recognized bioassay methodologies would also facilitate more precise environmental impact determination, particularly in waste import and export regulations.

## Figures and Tables

**Table 1:** Conditions of the ISO leaching methods of solid materials of soils.

Sample Preparation and Conditions		ISO		
		ISO/TS 21268-1 [22]	ISO/TS 21268-2 [23]	ISO/TS 21268-4 [25] ^a^
Sample	Particle diameter (Sieving)	4 mm	4 mm	4 mm
	Weight (g)	350 ± 5	90 ± 5	-
Solvent		1 mM CaCl_2_	1 mM CaCl_2_	1 mM CaCl_2_
Solid:liquid (W:V)		1:2	1:10	1:10
Leaching condition	Temperature (°C)	20 ± 5	20 ± 5	20 ± 5
	Time (h)	24 ± 0.5	24 ± 0.5	a: 4 h b: 40 h c: 4 h total: 48 h
	RPM	5–10	5–10	5–10
Filtration	Type	Membrane filter	Membrane filter	Membrane filter
	Pore size (µm)	0.45	0.45	0.45

^a^ The objective of the ISO/TS 21268-4 method considered the effects of pH in leachates.

**Table 2:** Conditions of the CEN leaching methods of wastes.

Sample Preparation and Conditions		EU			
		CEN ^a^ 12457-1 [26]	CEN 12457-2 [27]	CEN 12457-3 [28]	CEN 12457-4 [29]
Sample	Particle diameter (Sieving)	4 mm	4 mm	4 mm	10 mm
	Weight (g)	175 ± 5	90 ± 5	175 ± 5	90 ± 5
Solvent		Distilled water, demineralized water or deionized water	Distilled water, demineralized water or deionized water	Distilled water, demineralized water or deionized water	Distilled water, demineralized water or deionized water
Solid:liquid (W:V)		1:2	1:10	Step 1 1:2 Step 2 1:10 (cumulative)	1:10
Leaching condition	Temperature (°C)	20 ± 5	20 ± 5	20 ± 5	20 ± 5
	Time (h)	24 ± 0.5	24 ± 0.5	Step 1: 6 ± 0.5 Step 2: 18 ± 0.5	24 ± 0.5
	RPM	5–10	5–10	5–10	5–10
Filtration	Type	Membrane filter	Membrane filter	Membrane filter	Membrane filter
	Pore size (µm)	0.45	0.45	0.45	0.45

^a^ CEN European Committee for Standardization.

**Table 3:** Conditions of the standard leaching methods of wastes in US EPA.

Sample Preparation and Conditions		US EPA			
		EP [19] ^a^	TCLP [16] ^b^	SPLP [17] ^c^	MEP [18] ^d^
Sample	Particle diameter (Sieving)	9.5 mm	9.5 mm	9.5 mm	0.5–5 mm
	Weight (g)	100	100	100	100
Solvent	Heavy metal	Acetic acid (0.5 N) + distilled water → 2 L (pH 5 ± 0.2)	Acetic acid 5.7 mL + reagent water 500 mL + NaOH (1 N) 64.3 mL → 1 L (pH 4.93 ± 0.05)	H_2_SO_4_:HNO_3_ (6:4) + reagent water (pH 4.2 ± 0.05)	H_2_SO_4_:HNO_3_ (6:4) + distilled water (pH 3.0 ± 0.2)
	Cyanide (CN)	-	-	Distilled water	-
Solid:liquid (W:V)		1:20	1:20	1:20	1:10
Leaching condition	Temperature (°C)	20~40	23 ± 2	23 ± 2	20–40
	Time (h)	24	18 ± 2	18 ± 2	24 (9 times extraction)
	RPM	Sufficient agitation	30 ± 2	30 ± 2	-
	Shaking width (cm)		-	-	-
Filtration	Type	Membrane filter	GFF (Glass fiber filter)	GFF	-
	Pore size (µm)	0.45	0.6–0.8	0.6–0.8	-

^a^ Extraction Procedure. ^b^ Toxicity Characteristic Leaching Procedure. ^c^ Synthetic Precipitation Leaching Procedure. ^d^ Multiple Extraction Procedure.

**Table 4:** Conditions of the standard leaching methods of waste and soil in Korea and Japan.

Sample Preparation and Conditions		Korea	Japan
		Waste Official Test Standard [21]	JLT-13 (Japanese Standard Leaching Tests No. 13) [47]
Sample	Particle diameter (Sieving)	0.5–5 mm	0.5–5 mm
	Weight (g)	100	100
Solvent	Heavy metal	HCl + distilled water (pH 5.8–6.3)	HCl + NaOH (pH 5.8–6.3)
	Cyanide (CN)	-	-
Solid:liquid (W:V)		1:10	1:10
Leaching condition	Temperature (°C)	15–25	20
	Time (h)	6	6
	RPM	200	200
	Shaking width (cm)	4–5	4–5
Filtration	Type	GFF	GFF
	Pore size (µm)	1	1

**Table 5:** Standard methods and threshold values for characterizing the ecotoxicity of wastes suggested by European countries and previous studies.

Bioassay (Test Methods)	Test Duration	Country or Organization				
		Czech (%) [13]	France (%) [12]	Germany (%) [14]	Italy (%) [15]	ISO/DS 17616 [68]
*Aliivibrio fischeri* (ISO 11348-3) [48]	15–30 min	EC_50_^a^ 1	EC_50_ 10	EC_50_ 10	EC_50_ 10	LID 8 20% inhibition
*Brachionus calyciflorus* (ISO 20666) [49]	48 h	-	EC_20_^a^ 1	-	-	20% mortality 30% inhibition
*Desmodesmus subspicatus*/*Raphidocelis subcaptitata* (EN ISO 8692) [50]	72 h	-	EC_50_ 10	EC_50_ 10	EC_20_ 20	LID 4 25% inhibition
*Daphnia magna* (EN ISO 6341/ISO 10706) [51,52]	24, 48 h	EC_50_ 1	EC_50_ 10 EC_20_ 1	EC_50_ 10	EC_50_ 10	LID 4 20% inhibition
	21 d	-		EC_50_ 10	-	-
*Ceriodaphnia dubia* (ISO 20665) [53]	7 d	-	EC_20_ 1	-	-	20% mortality 30% inhibition
*Poecilia reticulata* (EN ISO 7346-2) [54]	4 d	EC_50_ 1	-	-	-	-
*Lemna minor* (ISO 20079) [55]	-	-	-	EC_50_ 10	-	25% inhibition
*Sinapis alba* [56]	3 d	EC_50_ 1	-	-	-	-

^a^ EC_50_, 50% effective concentration; EC_20_, 20% effective concentration.

**Table 6:** Standard methods and threshold values for characterizing the ecotoxicity of wastes, as suggested by European countries and previous studies.

Solid Waste	Leaching Method	Test Species for Leachate Bioassay	Reference
Bottom ash and slag	EN 12457-2	*Aliivibrio fischeri*, *Daphnia magna*, *Raphidocelis subcapitata*, *Ceriodaphnia dubia*	[33]
Coal fly ash	TCLP 1311, EN 12457-2	*Aliivibrio fischeri*, *Brachionus calyciflorus*, *Daphnia magna*	[71]
Ash from sewage sludge combustion	TCLP 1311, EN 12457-2	*Aliivibrio fischeri*, *Daphnia magna*	[34]
Boiler slag, thin sludge, waste petrol, and sewage sludge	EN 14735	*Aliivibrio fischeri*	[59]
Solid residue from co-pyrolysis of plastic and pine biomass	ISO/TS 21268-2	*Aliivibrio fischeri*	[30]
Char residue	ISO/TS 21268-2	*Aliivibrio fischeri*	[31]
Ash from the combustion of coal, meat, and bone meal	EN 12457-2	*Aliivibrio fischeri*, *Raphidocelis subcapitata*, *Daphnia magna*	[35]
Coal fly ash	EN 12457-3	*Aliivibrio fischeri*, *Raphidocelis subcapitata*, *Daphnia magna*	[71]
Plastic wastes, used tires, and pine forestry biomass	ISO/TS 21268-2	*Aliivibrio fischeri*	[32]
Photovoltaic panels	EN 12457-2	*Aliivibrio fischeri*, *Raphidocelis subcapitata*, *Daphnia magna*	[36]
Cigarette butts	EN 14735	*Aliivibrio fischeri*, *Daphnia magna*, *Raphidocelis subcapitata*, *Ceriodaphnia dubia*	[66]
Fire-retardant coating systems	CEN/TS 16637-2	*Raphidocelis subcapitata*, *Daphnia magna*, *Danio rerio embryo*	[67]
Green liquor dregs	EN 12457-2	*Aliivibrio fischeri*, *Raphidocelis subcapitata*, *Lemna minor*, *Daphnia magna*	[37]
Weathered coal fly ash	EN 12457-2	*Aliivibrio fischeri*, *Raphidocelis subcapitata*, *Lemna minor*, *Daphnia magna*	[38]
Thermal conversed sewage sludge	EN 12457-2	*Aliivibrio fischeri*	[39]
solar cell panels (perovskite and silicon solar cells)	TCLP 1311	*Danio rerio*, *Daphnia magna*	[72]
Waste foundry sand	EN 12457-2	*Aliivibrio fischeri*, *Raphidocelis subcapitata*, *Daphnia magna*	[40]
Weathered incineration bottom ash	EN 12457–2	*Aliivibrio fischeri*, *Raphidocelis subcapitata*, *Lemna minor*, *Lepidium sativum*, *Daphnia magna*	[41]
Incineration bottom ash	EN 12457–2	*Aliivibrio fischeri*, *Raphidocelis subcapitata*, *Lemna minor*, *Daphnia magna*	[42]
Car fluff, fly ash, and sludge	EN 12457–2	*Aliivibrio fischeri*, *Daphnia magna*, *Raphidocelis subcapitata*	[43]
Sc-containing acid liquid waste	Liquid state	*Aliivibrio fischeri*, *Daphnia magna*	[73]
Slags from iron and steel industry production	EN 12457-2	*Aliivibrio fischeri*, *Daphnia magna*, *Sinapis alba* L., *Eisenia andrei*	[44]
Ash from calcium-rich fuel combustion	EN 12457-4	*Aliivibrio fischeri*, *Daphnia magna*	[74]

**Table 7:** Proposed concentrations of contaminant chemicals in wastes suggested by the US EPA and the Ministry of Environment in Japan and the Korean ministry.

Toxicity Characteristics					
Contaminants (mg/L)	TCLP ^a^	Federal Hazardous Waste Regulations	Effluent Standard		Waste Official Test Standard
	US EPA [16]	Canada [45]	Japan [47]		Korea [21]
		Effluent Release Limit	Municipal Solid Wastes	Industrial Wastes	
Antimony	-	0.3	-	-	-
Arsenic	5	-	0.1	-	3
Barium	100	2.5	-	-	-
Benzene	0.5	-	0.1	-	-
Cadmium	1	0.1	0.1	0.01	0.3
Carbon tetrachloride	0.5	-	-	-	-
Chlordane	0.03	-	-	-	-
Chlorobenzene	100	-	-	-	-
Chloroform	6	-	-	-	-
Chromium	5	1	2	-	1.5
Cobalt	-	0.3			
Copper	-	0.3	3	-	3
Cresol	200	-	-	-	-
Cyanide	-	0.2	1	Not to be detected	1
2,4-D	10	-	-	-	-
1,4-Dichlorobenzene	7.5	-	-	-	-
1,2-Dichloroethane	0.5	-	0.4	0.004	-
1,1-Dichloroethylene	0.7	-	0.2	0.02	-
2,4-Dinitrotoluene	0.13	-	-	-	-
Endrin	0.02	-	-	-	-
Heptachlor	0.008	-	-	-	-
Hexachlorobenzene	0.13	0.02	-	-	-
Hezachlorobutadiene	0.5	-	-	-	-
Hexachloroethane	3.0	-	-	-	-
Iron	-	-	10	-	
Lead	5	0.3	0.1	0.01	3
Lindane	0.4	-	-	-	-
Manganese	-	1	10	-	
Mercury	0.2	0.01	0.005	0.0005	0.005
Methoxychlor	10	-	-	-	-
Methyl ethyl ketone	200	-	-	-	-
Molybdenum		1			
Nitrobenzene	2	-	-	-	-
Oil	-	60	-	-	5% (*w*/*w*)
Organic P	-	-	1	-	1
Pentachlorophenol	100	-	-	-	-
Polychlorinated Biphenyl	-	0.01	-	Not to be detected	
Pyridine	5	-	-	-	-
Selenium	1	0.1	0.1	0.01	-
Silver	5	-	-	-	-
Tetrachloroethylene	0.7	-	0.1	0.01	0.1
Toxaphene	0.5	-	-	-	-
Trichloroethylene	0.5	-	0.3	0.03	0.3
2,4,5-Trichlorophenol	400	-	-	-	-
2,4,6-Trichlorophenol	2	-	-	-	-
2,4,5-TP (Silvex)	1	-	-	-	-
Vinyl chloride	0.2	-	-	-	-
Zinc	-	0.5	5	-	
Etc.			Various	Various	-
Various					
Dichloromethane			0.2	0.02	
Carbon tetrachloride			0.02	0.002	
cis-1,2-dichloriethylene			0.4	0.04	
1,1,1-trichloriethane			3	1	
1,1,2-thricholoroethane			0.03	0.006	
1,3-dichloropropene			0.02	0.002	
Thiram			0.06	-	
Thiuram			-	0.006	
Simazine			0.03	0.003	
Thiobencarb			0.2	0.02	
Hydrogen ion concentration			5.8-8.6		

^a^ TCLP: Toxicity Characteristic Leaching Procedure.

## Data Availability

Not applicable.
